# Supplementary material for: Application of Covalent Organic Frameworks (COFs) as Dyes and Additives for Dye-Sensitized Solar Cells (DSSCs)
Source: Nanomaterials (Basel). 2023 Mar 28;13(7):1204. doi: 10.3390/nano13071204 (PMC10097336; doi:10.3390/nano13071204)
Supplement: Supplementary file 1 [file nanomaterials-13-01204-s001.zip › nanomaterials-2284159-supplementary.pdf]

Supplementary Information

Application of Covalent Organic Frameworks (COFs) as Dye and additives for Dye-Sensitized Solar Cells (DSSCs)

Diogo Inácio[a], Ana Lucia Pinto[a], Ana B. Paninho[a], Luis C. Branco[a], Sunny K. S. Freitas[a,b*], Hugo Cruz[a*]

[a]LAQV-REQUIMTE, Department of Chemistry, NOVA School of Science and Technology, NOVA University of Lisbon, Campus de Caparica, 2829-516 Caparica, Portugal.

[b]Instituto de Química—UFRJ Avenida Athos da Silveira Ramos, n° 149, Bloco A—7° andar Centro de Tecnologia—Cidade Universitária, Ilha do Fundão, Rio de Janeiro 21941-909, Brazil.

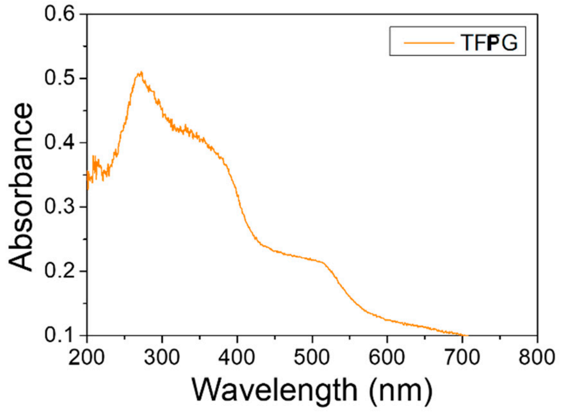

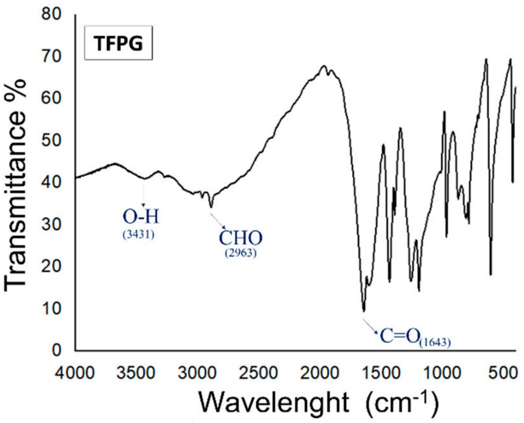

Figure S1. Diffuse reflectance (left) and FT-IR for TFPG.

-RIO-70

The synthetic procedure was adapted from literature[7] but some alterations were performed to go into the encounter to our goals. In a reactor (high pressure vessel, ChemGlass, 48 mL) were placed 0.30 mmol of Hydrochlorinated Pararosaniline (Scientific Exodus) and 0.35mmol of synthesized TFPG, and also add as solvent 12 mL of 1,4-Dioxane (99.5% Merck) and 3 mL of 6 M of acetic acid (99.7% Honeywell) in this order. Leaving the reactor slightly open, the reaction was placed in an oil bath at 120ºC for 10 minutes with agitation. After the reaction time 2 photoanodes (only with blocking layer and TiO_2 layer) were placed inside the reactor and the reaction continued for more than 3 days without agitation in a closed reactor.

When the reaction ended, the glasses were removed, washed with ethanol and left to dry in a dark environment. After drying, assembly and testing were carried out with RIO-70 replacing the dye.

The rest of the solid was vacuum filtered on a Büchner funnel with filter paper and washed with ethanol (two layers of filter paper was used due to the possibility of losing material through the filter pores). The resulting solid is "packaged" in a small envelope of filter paper, and left in a soxhlet with ethanol, until it stops releasing the precursors into the solvent (at this point the COF pores are filled with ethanol).

To dry the final solid, it was sent for supercritical CO2 drying. After this step, the resulting powder has a dark wine color in addition to having empty pores (air) and can be characterized.

Previous characterization (Freitas at al. 2021) by UV-visible, infrared and BET spectroscopy (Brunauer-Emmett-Teller-this method is used to analyze surface area and pore size of powder material through N2 absorption), as published[7].

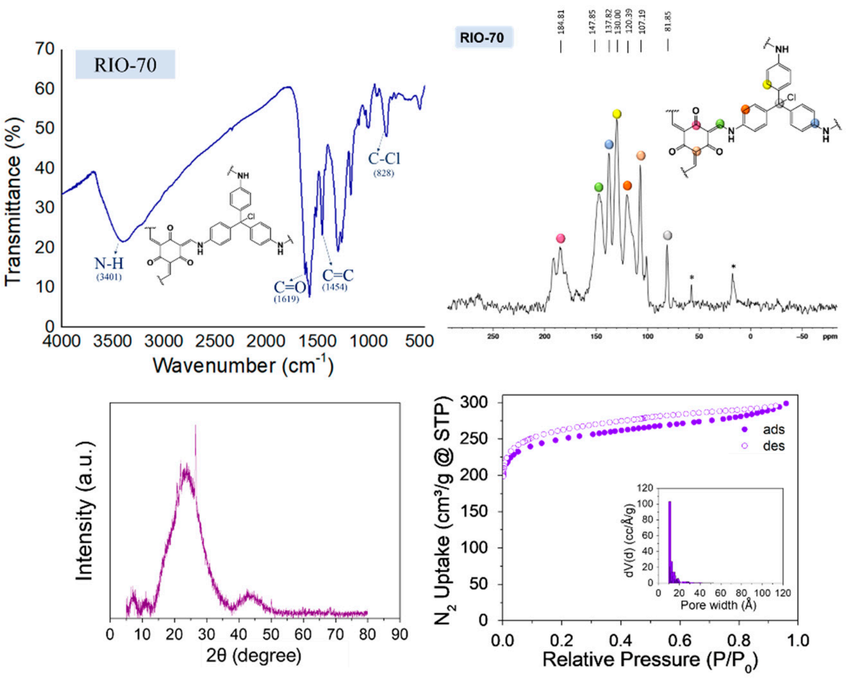

Figure S2 – Characterization for RIO-70.

-RIO-43

First was reproduce the synthesis of the compound as previous described in the literature by Freitas et al. 2020.[6] A first reaction only for the formation of the product (bulk) and, the following ones, with deposition on the photoanode.

The synthetic procedures of the compound were adapted from the described in literature[6] with slight changes: 0.30 mmol of Thionine acetate (85% Sigma-Aldrich) and 0.55 mmol of TFPG were weighed and placed inside the reactor. Then added 12 mL of 1,4-Dioxane and 3 mL of 6M acetic acid as solvent in that order. Leaving the reactor slightly open. The reaction was left in an oil bath at 120°C for 10 minutes with stirring. After this time, 2 photoanodes (blocking layer and TiO_2 layer) were placed inside the reactor and the reaction continued for another 3 days, now without agitation and with the reactor completely sealed.

After drying, assembly and testing was carried out with RIO-43 replacing the dye.

The solid resulting from the reactions was filtered by vacuum in a Büchner funnel with filter paper washed with ethanol. The resulting solid is “packed” in a small envelope of filter paper and left in a Soxhlet with ethanol until it stops releasing dye into the solvent.

To dry the final solid, it was sent for supercritical CO2 drying. After this step the resulting powder, which had a rather dark color, could be characterized.

The previous characterization[6] by UV-visible, infrared and BET spectroscopy.

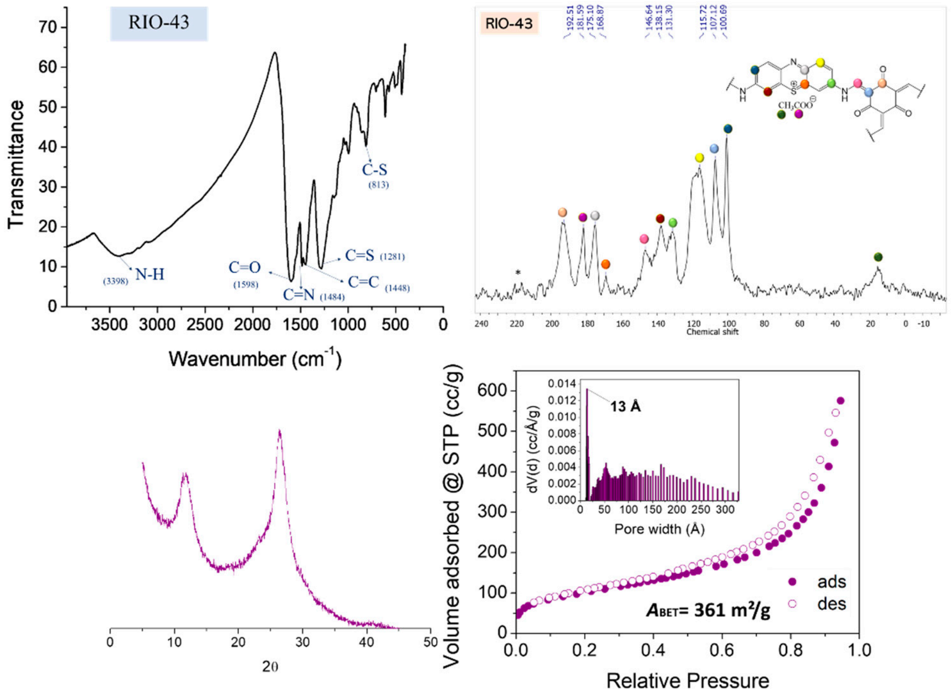

Figure S3. Characterization for RIO-43.

-RIO-55

First was reproduce the synthesis of the compound as previous described in the literature by Freitas et al. 2020.[6] The procedure was slightly different from the previous described, while the last one will be mentioned in a next experimental step.

The synthetic procedures of the compound were carried out as described in the literature [6] with slight changes in order to insert the photoanodes in the reaction: 210 mg (0.5 mmol) of Bismarck Brown Y (50% Sigma Aldrich) and 130 mg (0.5 mmol) of TFPG were weighed and added to the reactor. As a solvent, 13 mL of 1,4-dioxane and 3 mL of 6M acetic acid were added.

After this step, the anode (blocking layer and TiO_2 layer) was immediately inserted and the reactor was left in an oil bath at 120ºC, with stirring and closed for 3 days.

At the end of the reaction, the anode was collected, washed with ethanol and left to dry in an environment protected from light. When dry it was mounted in a cell where RIO-55 took on the role of dye replacement.

The solid obtained at the end of the reaction underwent the same filtration and drying process as the previous ones and had a reddish color.

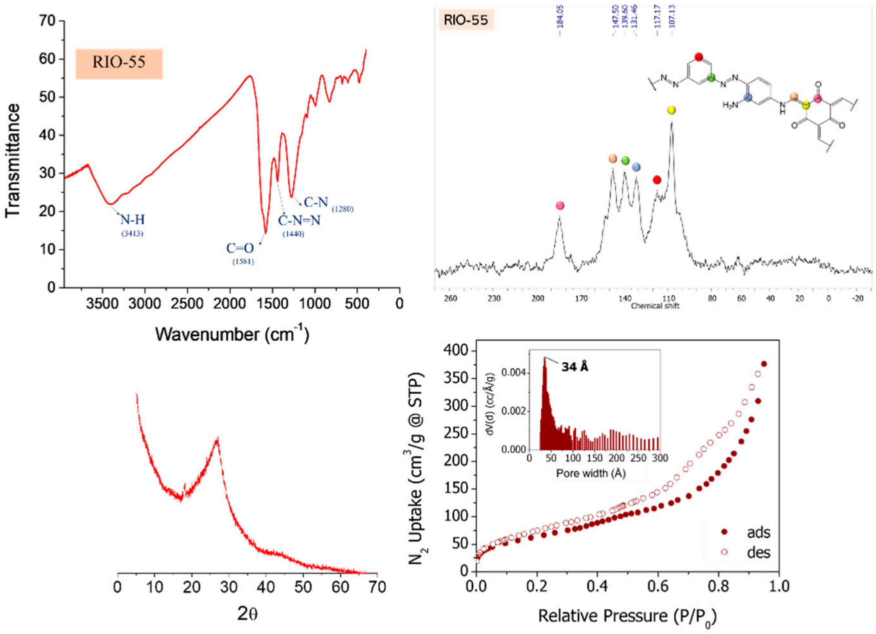

Figure S4. Characterization for RIO-55.

-COF DAAQ

The synthesis was performed followed the previously reported and characterized by DeBlase et al. Although this material has already been published, the synthesis procedure was developed using another methodology (with an excess of aldehyde), which we developed during the course of the work, in order to apply the COF to the photoanode: 140 mg (0.6 mmol) of DAAQ (97% Sigma-Aldrich) and 170 mg (0.8 mmol) of TFPG were weighed. These precursors were added to the reactor and 10 mL of 1,4-dioxane and 3 mL of 6 M acetic acid were added in that order. Finally, a photoanode (with blocking layer and TiO_2 layer) was added to the reactor, closed and the reaction was carried out in oil at 120ºC, with stirring for 3 days. At the end of the reaction, the glass was removed, washed with ethanol and left to air dry in a dark environment. After drying, a cell was assembled and tested with this anode in which the COF replaces the role of the dye. The COF resulting from the reaction was filtered and dried like the previous ones, getting a brownish color. The results of the reported characterization[21].

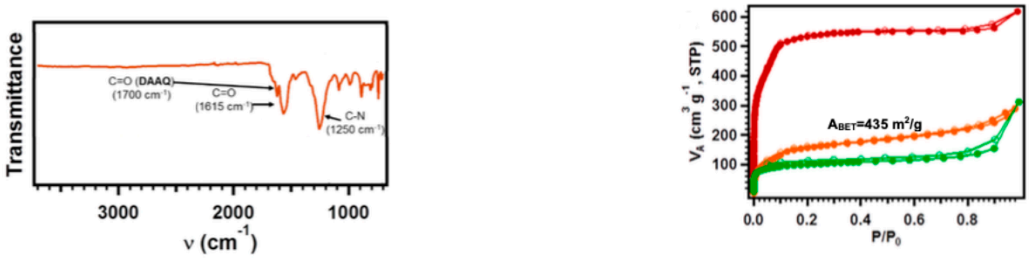

Figure S5- COF DAAQ-TFP a) FTIR b) BET orange spectra.
